# Policy and regulatory measures supporting the implementation of nature-based solutions in urban stormwater management of private properties: Insights from Finland

**DOI:** 10.1007/s13280-025-02253-2

**Published:** 2025-09-27

**Authors:** Aino Saarinen, Piia Leskinen, Aleksi Reini, Nora Fagerholm, Elina Kasvi

**Affiliations:** 1 Department of Geography and Geology, University of Turku, Vesilinnantie 5, 20014 Turku, Finland; 2 Energy and Environmental Technology, Water and Environmental Engineering, Department of Sustainable Environment, Faculty of Engineering, Turku University of Applied Sciences, Joukahaisenkatu 3, 20520 Turku, Finland

**Keywords:** Bottlenecks, Nature-based solutions, Private land, Stormwater management, Stormwater regulation, Water policy

## Abstract

Despite widely existing consensus that nature-based solutions (NbS) provide competitive and sustainable alternatives for grey infrastructure in urban water management, the implementation of NbS is lagging behind current needs. This study explores if and how regulatory measures can promote NbS adoption on privately owned land. Policy document analysis of Finnish stormwater programmes gathers how NbS are considered at municipal-level, key informant interviews aim to identify bottlenecks in stormwater regulations, and building permit analysis evaluates whether policy instruments have increased NbS implementation on private land. Results reveal fragmented municipal regulations due to a lack of national consensus, leading to bottlenecks for implementation such as limited knowledge, commitment, and institutional coherence. While current regulations have increased solution diversity, widespread NbS implementation requires mandating and consistent regulations aligned with sustainability goals, improved understanding of NbS functionality, examples and education, national cooperation, and recognition of NbS as viable stormwater management options.

**Supplementary Information:**

The online version contains supplementary material available at 10.1007/s13280-025-02253-2.

## Introduction

Stormwater systems can be considered as critical components of the urban water sector (Santos et al. 2019), yet their performance is increasingly compromised by the impacts of climate change. Intensifying extreme weather events and increased precipitation have reduced the capacity of many existing systems to meet urban water management needs. In response, climate change adaptation (CCA) objectives are increasingly incorporated into stormwater management and urban planning policies to increase sustainability and resilience (Zolina 2012; Henstra et al. 2020). However, improving conventional underground grey infrastructure is often prohibitively expensive, making it difficult to respond to climate change and CCA objectives. This has created a pressing need for complementary approaches that can strengthen and adapt existing systems.

Expanding green infrastructure (GI) and employing nature-based solutions (NbS) has been increasingly seen as the future solution for climate sustainable city development while also preserving biodiversity and ecosystems (Cohen-Shacham et al. 2019; de Oliveira et al. 2022; Corgo et al. 2024). The European Commission defines NbS as “actions which are inspired by, supported by or copied from nature” and they help to sustainably address environmental, social, and economic challenges (European Commission 2015). However, NbS are discussed in many different contexts, where the use of the term and solutions vary significantly (Sarabi et al. 2019). In urban environments, combinations of grey and green solutions are common, due to their effectiveness as well as space and cost restrictions in urban constructions (Ruangpan et al. 2020; de Oliveira et al. 2022). Implementation of decentralised NbS in private properties is beneficial for urban water management as they provide water retention and detention near to the origin, which prevents municipal stormwater systems from overloading, thus reducing the risk of urban floods (Zölch et al. 2017; Kumar et al. 2021). In addition, NbS have the potential to provide additional ecosystem services which makes them even more valuable for urban development (Eggermont et al. 2015).

Coherent policy frameworks and national-level legislation are often considered essential for mainstreaming NbS (Van den Ende et al. 2023). In efforts to renew water management planning to meet contemporary quality, quantity, and sustainability standards, many countries have developed national, ecosystem-based regulatory concepts. These include Best Management Practices (BMPs), Best Available Techniques, Low Impact Development, Water Sensitive Urban Design, Integrated Stormwater Management, River Basin Management Plans, and Sustainable Drainage System policy statements (Ellis and Lundy 2016; McDonald and Naughton 2019; Henstra et al. 2020; Jensen et al. 2020; Ruangpan et al. 2020). While existing frameworks provide direction for NbS implementation, they often lack clear guidance on choosing the most suitable approach for a specific context (Corgo et al. 2024).

Policy and regulations have been stated to have a key role in directing the stormwater management towards NbS (Geyler et al. 2019; Novaes and Marques 2022, 2023). Municipal-level regulations, such as land use planning regulations and water quality regulations, have been seen to have a potential in increasing the number of NbS implementation in water management also in private properties. In fact, binding municipal regulations have successfully increased private investments in green infrastructure (Mandarano and Meenar 2017). For example, planning regulation tools such as the biotope area factor (BAF) and its later developed versions can promote sustainable water management (Kopetzki and Hausmann 2016). Modelled scenarios have also demonstrated that municipal-level regulation is the most effective approach in terms of environmental outcomes. Binding regulation at the municipal-level can reduce waterborne emissions more effectively than commonly used methods such as fee- or penalty-based systems (William et al. 2017). In contrast, cases where regulation is based on voluntariness have been viewed as problematic, as they have not led to an increased adoption of NbS (Venuti et al. 2025).

Despite the well-known issues in stormwater management systems and institutional efforts, such as policy and regulations for increasing the number of sustainable alternatives, the traditional grey infrastructure, such as pipes and underground storage structures, are still often chosen over nature-based alternatives. Previous studies have identified various political, institutional, and financial barriers for implementation of NbS in urban stormwater management, such as lack of know-how, path dependency, cultural barriers, and inadequate or insufficient regulation (Johns 2019; Santos et al. 2019; Sarabi et al. 2019; Khalaji et al. 2025). In addition, the ambiguity of roles and responsibilities and lack of collaboration between different sectors (sectoral silos) and different levels of land use planning have been identified as significant barriers in several studies (Sarabi et al. 2019; Dorst et al. 2022; Venuti et al. 2025). The new stormwater management regulations and implementations concerning NbS have caused confusion regarding the division of responsibilities for stormwater issues and systems (Ardren and Davies 2023; Venuti et al. 2025).

Private landownership is commonly cited as the limiting factor in extensive NbS implementation (Johns 2019; Venuti et al. 2025). The motivation of private individuals to participate in stormwater management has been studied at the community level in small housing cooperatives and among individuals. In these cases, motivation has often stemmed from factors such as harm prevention (Ardren and Davies 2023). However, the choice of stormwater management solutions in new residential buildings is typically carried out by the property developer that will sell the apartments and has no direct long-term interests on the property. This is where stormwater regulations play a crucial role, as they directly target new buildings and developers by mandating specific standards and measures.

Although efforts have been made to improve stormwater-related regulations, empirical evidence on how well these regulations function in practice remains limited. This study examines the relationships between policies, regulatory measures, and the grassroots-level implementation of NbS in urban water management in private properties in Finland. Finland follows EU-wide environmental directives but still lacks an up-to-date national regulatory framework for stormwater management and NbS, therefore presenting a relevant case of decentralised municipal governance, fragmented local regulation. The Finnish Land Use and Building Act delineates responsibilities for traditional stormwater management, but it fails to adequately address the responsibilities for NbS (Venuti et al. 2025). We seek to illustrate how regulatory uncertainty and other barriers from municipal-level policy to grassroots-level affect implementation of NbS in water management.

Due to the lack of national-level regulation, Finnish municipalities apply varied regulatory approaches, underlining the need for context-specific analyses and positioning Finland as a valuable case study for investigating local level challenges and potential pathways in mainstreaming NbS. Prior research has not sufficiently addressed the impact of fragmented municipal regulations on private landowners’ motivation to implement NbS. While some attention has been paid to municipal decision-making and regulation, the implementation and effects of these regulations have not been examined within the broader decision-making and regulatory landscape.

Although previous studies have identified regulatory shortcomings and inconsistencies, further research is needed to specifically assess regulatory mechanisms and understand the connections between decision-making processes, regulations, and practical implementation. Previous studies do not sufficiently assess the effectiveness of regulations at the practical level. This study consists of three parts: First, we conduct a policy document analysis of stormwater strategies to examine how international and national policies of NbS are considered and implemented at municipal-level in Finland. Second, with key informant interviews, we identify significant bottlenecks in current stormwater regulations inhibiting the NbS implementation locally. Third, by utilising building permit analysis, we examine if regulation instruments favouring NbS succeeded in increasing the implementation of NbS on privately owned land.

## Study area and context: Policy and regulatory landscape

### Study area

Finland is a Nordic country with population of about 5.6 million. Annual precipitation averages 600 mm, but comparison of climate normal periods 1961–1990 and 1991–2020 reveals an increasing precipitation trend (Lintunen et al. 2024). The annual mean temperatures have simultaneously risen by approximately 1.3 °C to a current range of − 2 to + 5 °C, reflecting the climate change together with short-term variability patterns. Urbanisation has steadily increased, with 85.77% of the population living in urban areas in 2023, primarily in the south. This has led to typical urban challenges such as densification, loss of green space, and more impermeable surfaces (Ala-Mantila et. al. 2023). Finland is a good example of a country with fragmented stormwater legislation is one where regulations are built on older frameworks, with reforms driven by the EU, and where local actors (i.e. municipalities and private landowners) bear significant responsibility for organising stormwater management (Venuti et al. 2025). Stormwater and NbS are governed by scattered and often non-binding regulations, primarily consisting of recommendations rather than enforceable mandates.

**Fig. 1:**
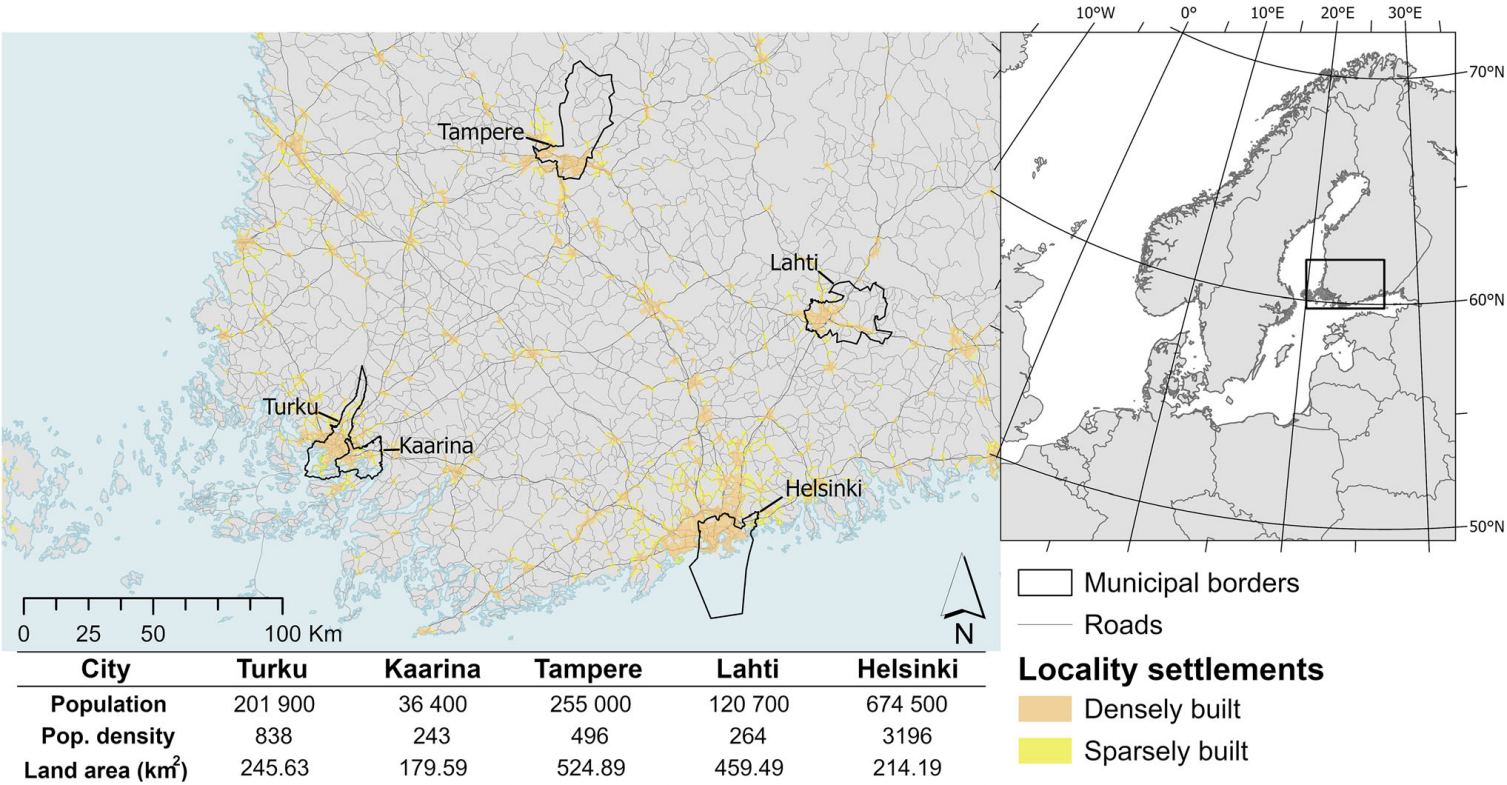
Location of the studied cities along with their populations, land areas and population densities. First and second parts of this study focus on all five cities, while the third part examines Turku located in Southwest Finland. In the Finnish context, Turku is classified as a large city

This study focuses on five cities in Southern Finland, Turku, Kaarina, Tampere, Helsinki and Lahti (Fig. 1) that serve as an example of stormwater policy and regulations in Finnish cities. The cities were chosen because they can be considered as frontrunners in stormwater management, and thus, they should have the most up-to-date and advanced regulations currently available in Finland for green infrastructure, stormwater management, and the implementation of NbS. The selection of cities was also strongly based on cities ambitiousness towards environmental protection and sustainability goals in city strategic level. The selected cities represent a diverse range of southern Finnish municipalities in terms of area, population size, and urban density, while still being sufficiently large to justify the need for stormwater management solutions in urban areas (Fig. 1). At the same time, their differing needs and urban structures have led to the development of distinct operational principles, as each city has independently determined its own approaches to stormwater regulation and management. Their stormwater systems also vary by age and condition; some areas still use combined sewers, but all new systems are separated from wastewater, and stormwater is discharge untreated into local waterbodies. Together these cities account for approximately one-fourth of Finland’s total population, underscoring the considerable influence of their stormwater governance on a significant proportion of the national population.

### Water and NbS policy and regulation from EU to municipal level

**Fig. 2:**
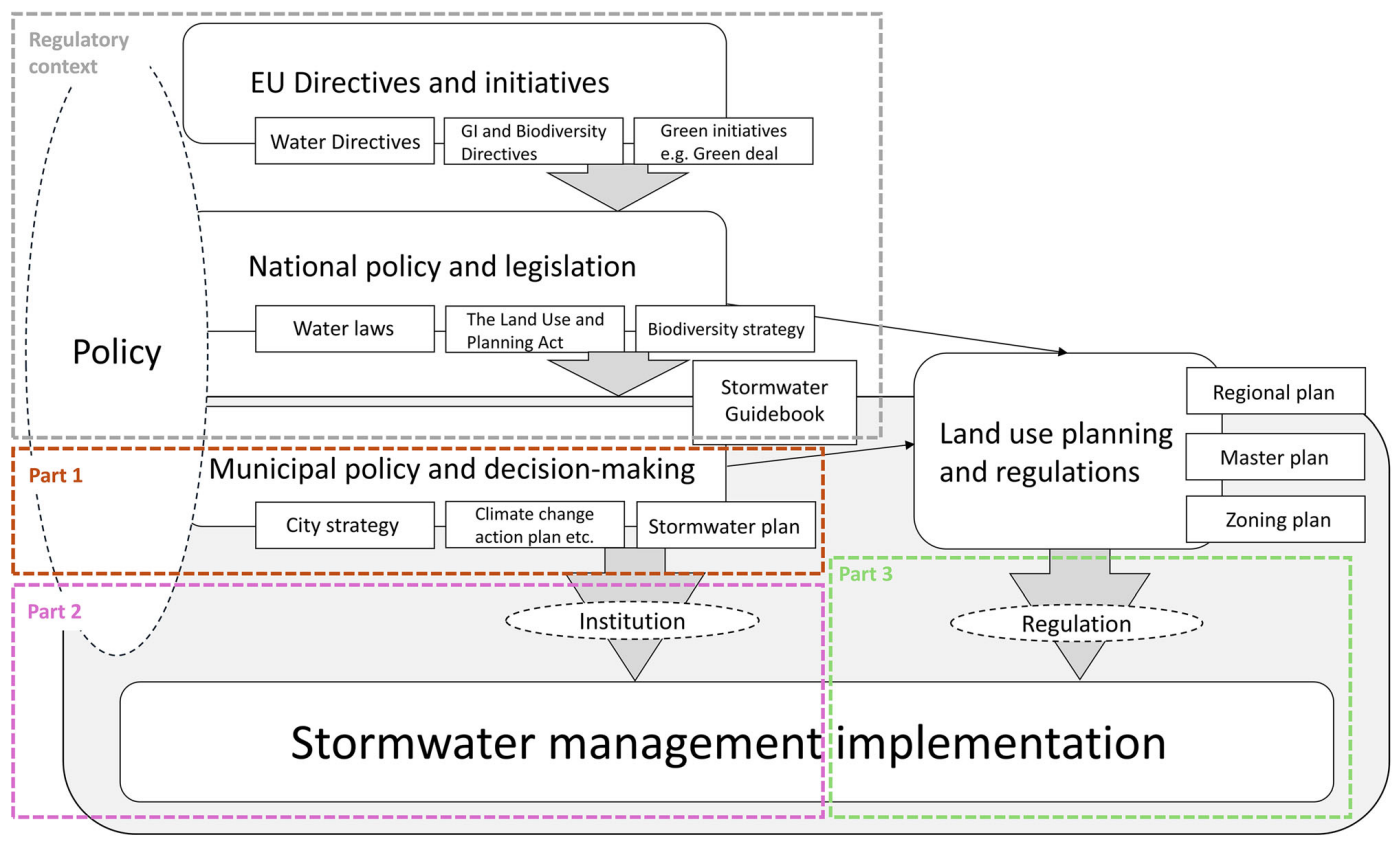
Hierarchical flow of the stormwater management from EU directives, via national policies (Regulatory context) down to land use planning municipal policies, ultimately reaching the stormwater management implementation. The three parts of this research (Part 1, 2, and 3 in the figure) are linked to different, critical parts of the stormwater management landscape and implementation

The EU decision-making is in a key position in directing water management and the application of NbS, as legislation in EU countries is formulated based on EU decisions (Fig. 2, Regulatory context). While there is no dedicated framework for stormwater, it is addressed indirectly within broader context of water directives such as the Water Framework Directive, the EU Floods Directive and the Urban Wastewater Treatment Directive. These directives emphasise pollution prevention in surface waters and proper wastewater treatment, including measures to regulate the quantity and quality of stormwater and address potential consequences (European Parliament 2000, 2007, 2024b). Flood risks are also widely considered, including urban floods (European Parliament 2007).

The EU has proposed several strategies promoting GI, NbS, and biodiversity in urban areas, including The European Green Deal complimented with initiatives like Green City Accord, Green Capital and Green Leaf, The EU Biodiversity Strategy, and The EU Green Infrastructure Strategy (European Commission 2013, 2019, 2020). In addition, the EU Nature Restoration Law introduces new regulations to expand green space in urban areas as the target is to ensure no net loss of green space by 2030 (European Parliament 2024a). NbS with similar policy-oriented concepts have been recognised and encouraged in water legislation with integration of protection and sustainable management into other water policy areas, protection of aquatic ecosystems, public involvement and consultation, and ecological status classifications (European Parliament 2000).

As EU directives do not set any requirements for mechanisms or tools for implementation, responsibility for actions is with the EU states. The Finnish water-related legislation and regulations comprise several laws: The Land Use and Building Act, being the key legislation in Finnish land use management, The Water Act, The Water Service Act, and The Flood Risk Management Act where stormwater is identified as a potential risk in urban areas (FINLEX 2010). In Finland, the stormwater management has been traditionally included in the responsibilities of water utilities. This changed in 2014, when the laws on amending the Land Use and Building Act (682/2014) and the Water Services Act (681/2014) came into force and the responsibility of stormwater management was given to the municipality, except for private properties where the property owner is responsible (Association of Finnish Municipalities 2017). The renewed legislation stated that the municipality should designate a multi-member stormwater management body that may issue specific regulations on stormwater management. It was also stated that the municipality may introduce a stormwater fee and make specific stormwater plans in new development areas, if necessary. The general objective of stormwater management is listed as follows:

1. to develop the systematic management of stormwater, especially in the local plan area;
2. to infiltrate and detain stormwater at its source;
3. to prevent damages caused by stormwater to the environment and property, also considering long-term climate change; and
4. to promote the uptake of separated sewer systems in all areas (FINLEX 2014).

NbS for water management are only indirectly addressed in the Finnish water legislation, where it is required that planning conforms to the environment and landscape, ensures water protection, sustainability, provision of a water supply, and the characteristics of the water bodies together with nature conservation, landscape values, and recreational needs (FINLEX 1999, 2011). However, Finland has taken other actions to protect biodiversity and promote GI: an ongoing action plan The National Action Plan for the Conservation and Sustainable use of the Biodiversity in Finland 2013–2020 and a New Biodiversity Strategy, currently underway (Ministry of the Environment 2020; Ministry of the Environment 2024).

Current guidelines for stormwater management at the municipal-level are produced by The Association of Finnish Municipalities in the form of Stormwater Guidebook (Association of Finnish Municipalities 2012, 2017). Along the lines of The Land Use and Building Act amendment (FINLEX 2014), it has set as a primary objective to prevent the formation of stormwater and reduce its volume through retention and infiltration systems as much as possible, before being directed to stormwater pipelines and receiving waterbodies. To reach the stormwater principles, the guidebook encourages Finnish municipalities to draft a stormwater programme that serves as a framework for visions, principles of operation, and a timeframe for implementing actions. The programme can be used as a framework for developing local stormwater plans, city planning, and for guiding building inspection.

**Fig. 3:**
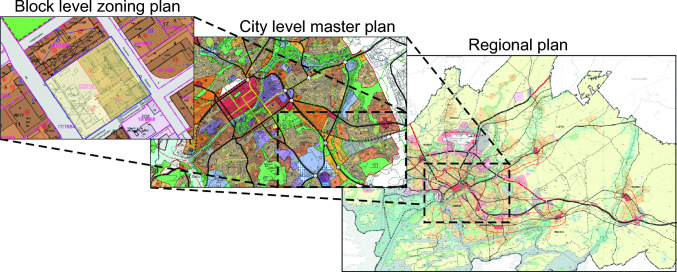
Zoning principles in Finland. Water management and planning are integrated in multiple scales from the broad framework of the regional plan to specific guidelines and regulations in the block-level zoning plan (modified from Leskinen et al. 2024)

Stormwater legislation and regulations have a strong connection to land use planning, governed by The Land Use and Building Act that outlines the actions to be taken at all levels of the Finnish zoning system (Fig. 3). In addition, land use is guided by National Land Use guidelines. Land use planning is performed in three spatial scales. The regional plan guides regional and cross-regional planning and has markings from current land use actions, and future development zones. A city-level master plan gives guidelines for land use and coordination at the municipality level. The block-level zoning plan gives detailed block-level instructions and guidelines for land use in public and private area. In addition to zoning, building code supports zoning at the municipal level and helps to preserve municipal characteristics. Together all three planning levels give a framework for stormwater management and development from detailed markings for implementations at the block-level to large-scale water management guidelines in the master plans of cities.

There are two common regulations stormwater management of private properties in Finland. The most widely applied regulation is the Hule-100 requirement which simply mandates one cubic metre of stormwater detention volume per 100 m^2^ of impermeable surface. The second regulation is blue–green factor (BGF), originally based on the widely known calculation tool biotope area factor (BAF) (Landschaft Planen and Bauen 1990). The Finnish version of the tool was developed by the city of Helsinki and refined during the iWater project in 2016–2018 (Helsinki 2016). BGF was initially designed to promote GI in new developments, but it also contains stormwater-related elements. BGF promotes the use of NbS by encouraging the use of permeable materials, enhancing GI, and increasing natural water retention capacity. Especially in cities, where BGF is tied to the Building Ordnance it has shown potential for increasing NbS implementation in private properties (Venuti et al. 2025). So far, BGF approaches are primarily used larger cities, whereas Hule-100 has been adopted all over Finland. BGF regulation is implemented via BGF calculation tool and the resulting coefficient. Regulations are targeted to private constructors with development projects in city areas. Municipalities set the regional BGF target values, and private developers include a BGF table and report in their building permit applications. The actual construction is carried out by private firms following building plans and permits, approved by the city.

## Materials and methods

### Document analysis of stormwater strategies

Document analysis of the stormwater strategies of five Finnish medium and large size cities was conducted to examine how international regulations of NbS are considered and implemented at municipal-level in Finland. Stormwater and NbS regulation methods were derived from municipal-level strategies, and stormwater programmes from the five studied cities (Table S1): Turku, Kaarina, Tampere, Lahti, and Helsinki. These documents are openly available on the websites of each city. The content of the stormwater programme of each city was thematically categorised representing goals for stormwater planning, sustainability, CCA, and NbS. Through categorisation, the contents of the stormwater programmes were compared with each other and in relation to municipal strategy, including city goals, future plans, and current regulatory frameworks. First-order thematic categorisation was carried out by identifying sentences containing the terms sustainability, CCA, or NbS to collect direct references to these topics. The documents were then searched again for related concepts, such as urban greening and biodiversity, that in practice, represent sustainability, CCA, or NbS implementation, in order to collect indirect references. Overall goals of the programmes were derived from the goals sections of the programmes and from the main text, if the sentence was clearly outlining the implementation of future goals.

### Key informant interviews to identify bottlenecks in NbS implementation

Semi-structured key informant interviews were conducted with stormwater experts from different sectors, cities, and levels of stormwater management to identify bottlenecks inhibiting the NbS implementation locally. Interviews sought to discover the interface between policymaking and practical implementation, with questions about commonly used regulations and their advantages and shortcomings (Table S2). The interview participants were recruited via email, contacting cities, planning organisations and companies working with urban planning, stormwater planning, and stormwater inspections at various organisational levels in selected Finnish cities, in local, regional, or national-level. Some were contacted through contact persons and personal organisational emails and some through company or organisation mailing list. The selection of participants was based on their willingness to take part. All willing participants, who were confident on their expertise in current stormwater regulations and use of NbS, were accepted to take part on the interviews regardless of the contact channel, organisation, position, background, or location. In total, 18 recruitment emails were sent, also hoping initial contacts to circulate the email. This led into seven interviews giving the initial response rate of approx. 38%. Interviews were conducted in the Autumn of 2023.

Interviewees represented three profession groups (Table 1): the building inspectors who were involved with block-level land use plans in city administration (interviewee 5), the zoning planners responsible for the development of the block-level land use plans (interviewees 1–4, 7), and the private sector planners, who designed the construction plans according to the regulations (interviewee 6). The interviewed experts worked primarily within the five selected cities, but some had connections to other Finnish cities as well, depending on their professional experience current work and background. Six out of five interviewees worked on public sector and five out of seven interviewees in municipal-level planning (Table 1). One interviewee worked in a private consulting company and had projects all over Finland and one worked in regional administration of Southwest Finland. This led into semi-unbalanced groups between public and private sector which may serve as a limitation in this study. Majority of the interviewees worked with municipal-level planning which serves well this study’s focus on municipal-level regulations, implementation, and bottlenecks. During the interviews, thematic saturation points were reached in the questions suggesting adequate total number of interviews.

**Table 1:** Interviewees sector and current position highlighting the representativeness and expertise of each interviewee

Interviewee	Current position	Sector
1	Senior planning officer	Public/Municipal
2	Senior stormwater advisor	Public/Municipal
3	Urban planner	Public/Municipal
4	Stormwater engineer	Public/Municipal
5	HPAC inspector	Public/Municipal
6	Senior planning officer	Private/Engineering/Consulting
7	Senior planning officer	Public/Regional

The goal was to understand advantages, bottlenecks, and improvement ideas concerning current stormwater regulation, from the perspective of increasing NbS. In addition, opinions and main bottlenecks for practical implementation of NbS were obtained from the interviews. All interviews were recorded and transcribed. Transcription was analysed and the answers were thematically classified using content analysis to find the relevant regulatory bottlenecks during different phases of planning. The bottlenecks were broadly classified into four categories following the existing literature about barriers in stormwater management, GI and NbS implementation: institutional and organisational barriers, engagement and uncertainty barriers, educational and knowledge barriers, barriers, and insufficient regulation (Johns 2019; Sarabi et al. 2019).

### Regulations from building permits

**Fig. 4:**
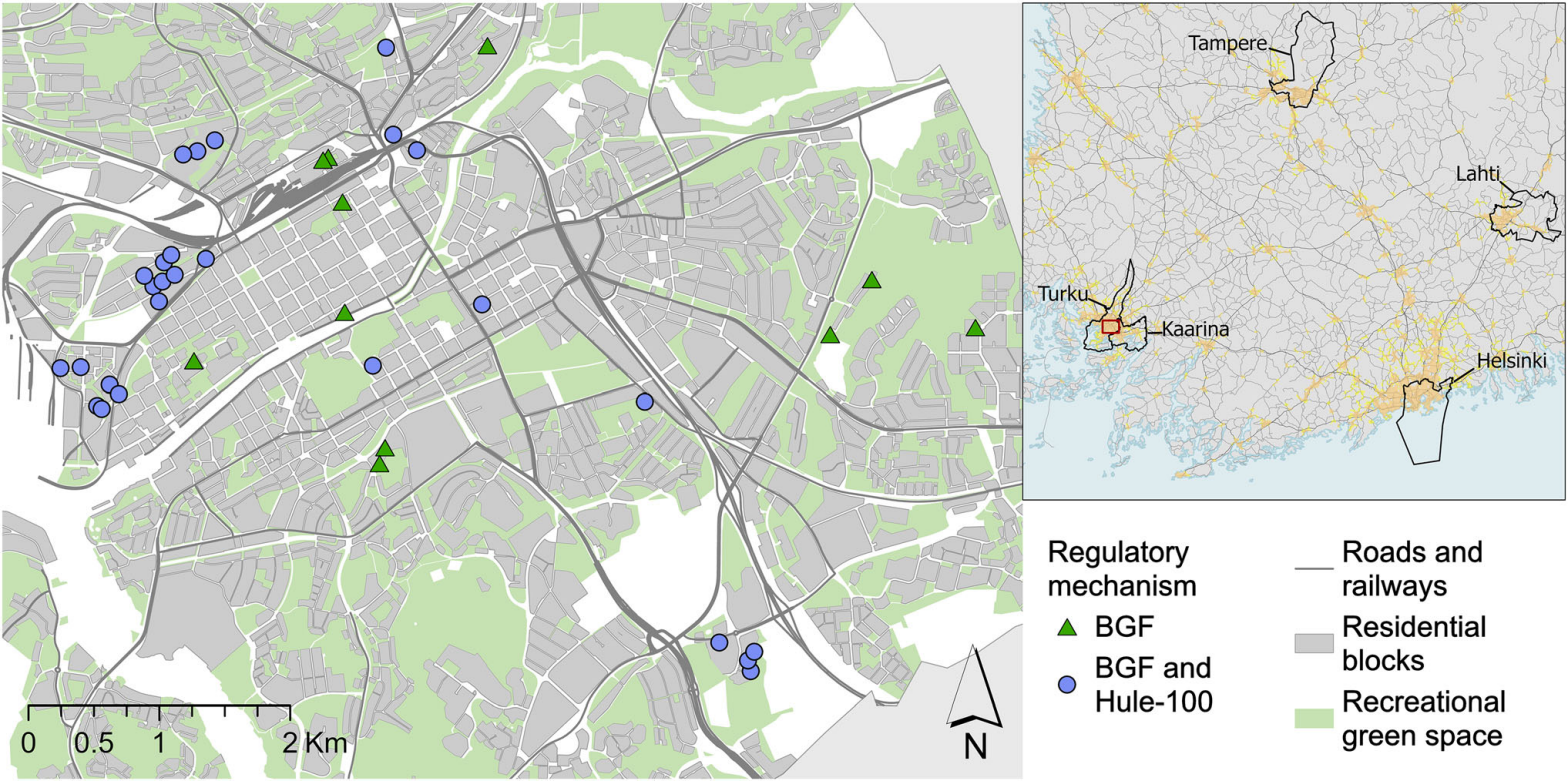
Locations and distribution of BGF and/or Hule-100 regulatory mechanism in 39 building permit sites in Turku

Building permits for new construction sites in Turku were analysed to evaluate the impacts of the applied stormwater regulations Hule-100 and BGF on the NbS implementation in the private properties. Building permits are public information, and they can be accessed by request. For this study, all the plans regulated by BGF were requested from the City of Turku. Suitable sites were selected based on the availability of the building permit documents, resulting in 39 urban sites near city centres being selected for the analysis (Fig. 4). These building permits were applied for during the years 2021 and 2022. To further investigate the impacts of BGF on NbS in stormwater management, several key indicators were obtained from the BGF calculation sheets. To quantify the BGF’s impact on the NbS implementation, percentage of each site’s lot area covered by buildings, green spaces, and impervious surfaces was calculated. This data along with BGF score and used stormwater management solutions were compiled in a worksheet and spatial dataset. In addition, planning maps and stormwater plans were individually inspected, and all water management related markings and interesting details were collected. The building permits included many separate documents, including a stormwater plan, a yard plan, a construction planning map, a plan description, and a BGF calculation sheet.

## Results

### Stormwater policy and regulations in the studied cities in Finland and their linkages to NbS

The emphasis on nature and the environment varies notably between the city strategies. All five cities acknowledge environmental goals, but CCA, NbS, and sustainability receive uneven emphasis with varying priorities. While water management is not featured in the city strategies, connection to nature and environmentally friendly actions have been recognised in each strategy (Table S1). Additionally, some city strategies (Turku, Tampere) mention Agenda 2030 mission and goals as base for strategy work. Notably, Turku, Lahti, and Helsinki have committed to the EU Green City Accord. Each city has also developed additional strategies for city development and climate change mitigation and adaptation.

Stormwater programmes complement other strategic documents by providing water management perspective. A dedicated stormwater programme recommended by the Association of Finnish Municipalities had been prepared in four of the five cities (Table 2). Stormwater programmes vary in focus: some emphasise improving governance and knowledge, while others prioritise water management measures to meet the sustainability, biodiversity, and GI objectives (Table 2). Some describe the current situation in detail and reflect the goals accordingly, while others focus more on strategic future goals. All plans include goals for urban flood management and water quality and quantity, and most also address organisational goals and knowledge enhancement. Some stormwater programmes address biodiversity, carbon neutrality, circular economy, and CCA. Ecosystem services provided by nature have been recognised and linked to well-being goals. Although most plans include CCA, sustainability, and NbS-related actions (Table 2), only one plan addressed them extensively. Nevertheless, the NbS goals of all the plans are ambitious, with local water retention and detention goals prioritised and NbS examples provided. The multiple benefits of NbS have also been recognised and mentioned in all plans. In addition, some current weak spots have been recognised in the plans and mentioned in the future development goals. It is worth noting that the financing of stormwater systems differs between the cities. Some include stormwater system maintenance and investment costs in the city budget, and others collect a separate stormwater fee.

**Table 2:** Comparison of studied cities, strategies, and stormwater management planning

City	Turku	Kaarina	Tampere	Lahti	Helsinki
	*Current stormwater policy and regulations*				
Regulations	Hule-100 and BGF	Hule-100, BGF alternative	By modified Hule-100, BGF	Hule-100	Hule-100 and modified BGF
Extend	New and infill developments in private plots	Some newly developed private plots. BGF alternative currently not in active use	Some newly developed private plots on dense areas	Some newly developed private plots	Some newly developed private plots
Regulatory enforceability	Mandating BGF target value, recommendation for Hule-100	Mandates permeable surface. No Hule-100 provisions	Mandating regulations are set plot specifically	Case specific mandating detention requirement (Hule-100 or other)	Mandates BGF whenever possible, BGF includes Hule-100
Building code	Prioritises on-site stormwater treatment and retention, recommends Hule-100. Sets targets for BGF	Provision mandating the primary on-site stormwater infiltration. If not possible, conduction to open conduction systems or stormwater sewers. One third of the plot must be permeable	No mixing of wastewater and stormwater. Recommends on-site stormwater infiltration	Provision mandating the primary on-site stormwater infiltration. If not possible, conduction to stormwater system without causing harm	No wastewater and stormwater mixing. Primary on-site stormwater infiltration. Prioritises above ground nature-based stormwater structures. Mandates BGF inclusion
Financing of stormwater management	General budget, no separate stormwater fees	General budget, no separate stormwater fees	Stormwater fee based on plot and building characteristics, paid by plot owner	General budget, no separate stormwater fees	General stormwater fee, paid by plot owner
	*Stormwater programme*				
Published	2016	No stormwater programme	2023	2011	2018
Content	Outlines future goals for stormwater management	–	Reviews current situation and outlines future goal	Reviews current situation and outlines future goals	Outlines future goals for stormwater management
Focus	Capacity building, enhancing water quality, urban flooding, improving preparedness	–	Enhancing water balance and biodiversity, water quality and quantity, urban floods	Enhancing of stormwater management, water quality and quantity, urban floods, biodiversity, information exchange	Capacity building, localised stormwater management, water quality and quantity, climate change, biodiversity
CCA and sustainability	Mentions climate change and posed challenges in the introduction. Indirectly addresses sustainability and CCA measures such as urban floods prevention and water quality goals	–	Addresses challenges posed by climate change and discusses climate resilience and CCA. Mentions sustainability and CCA measures. Encourages to comprehensive planning,	Mentions challenges posed by climate change. Indirectly refers sustainability and CCA with topics such as urban floods prevention and water quality and future goals	Extensively addresses challenges posed by climate change. Recognises climate resilience and CCA as a strategic goal and promotes CCA and sustainability measures in stormwater management
NbS	Promotes NbS in building processes, incl. green roofs, and stormwater retention and detention. Recognises benefits such as ecosystem services	–	Promotes NbS and multipurpose of stormwater structures, and stormwater retention and detention	Promotes NbS with examples, and stormwater retention and detention	Promotes stormwater retention and detention, and protection of existing natural environments and recognises benefits such as ecosystem services

Based on the document analysis (Table S1), the regulation of stormwater management varies between the cities, with differing applications of Hule-100 and BGF and varying integration of CCA and NbS. In all cases, regulation relies at least partly on voluntariness. All cities use some version of Hule-100 rule, and it is often included as a recommendation or applied with regional conditions. In Turku, the recommendation of the use of Hule-100 is ruled by the building code and can vary depending on the location. Lahti takes a less systematic approach, setting detention requirements by planning area characteristics and applying Hule-100 only in some cases. The basic version is also used in Kaarina and Helsinki to some extent. Hule-100 traditionally only considers the detention capacity per area of sealed surface; however, in recent developments, NbS and CCA goals have been included. Tampere has developed its own variations of Hule-100, which are mandatory in certain locations. New versions set the climate-sensitive detention requirement of 1.1 m3 (Hule-43) and a minimum share of stormwater that should be detained by a raingarden (Hule-51).

Four out of five cities use a BGF of a similar alternative (Table 2). Turku has included a BGF requirement in the building code and requires the use of BGF in any case, even when adding new buildings into existing city structure. Some exceptions within the BGF score are allowed, but generally, the target value is dependent on land use. Tampere BGF has been applied to zoning plans in densely built and nature-sensitive areas, with the target level varying by land use. Kaarina uses a distinct stormwater calculation factor tool that focuses directly on permeable and impermeable surfaces, retention, detention, and reuse. Unexpectedly, the use of this tool has recently been limited.

### Advantages, bottlenecks, and improvement ideas for NbS in stormwater policy, regulation, and implementation

Regulations can promote permeable surfaces and support NbS and urban greening, but they currently face barriers of policy incoherence, limited expertise, poor communication, and space constraints. In the key informant interviews, the experts mentioned several ways in which current stormwater regulations support the increase of NbS. They pointed out that stormwater regulation is generally well accepted, and the need for regulation is recognised. Both Hule-100 and BGF encourage water-sensitive urban planning with more permeable surfaces. Among the interviewees, Hule-100 rule was favoured for its simplicity and easy-to-understand construction and results. Hule-100 was evaluated to have a greater impact because it has become popular all over Finland. The BGF tool was valued for increasing green areas and integrating GI in new constructions, encouraging urban greening, and addressing ecological consideration. It supports CCA and increases transparency in zoning and planning processes.

The identified bottlenecks in regulation ranged from the themes of policy and BGF features to planning and monitoring of the implementation (Table 3). The interviewees identified several bottlenecks that inhibit the use of NbS in stormwater management. The identified bottlenecks were mostly related to 1) lack of coherence in policy, 2) shortcomings of the current stormwater regulations, 3) lack of know-how on NbS, and 4) organisational and structural challenges (Table 3).

**Table 3:** The most significant bottlenecks in stormwater regulation for NbS implementation and best practices identified by stormwater experts (modified from Leskinen et al. 2024 p 33–35)

*Barriers*	*Recommended best practices*
1. Lack of coherence in policy level	
*1.1 Lack of CCA plans (3 mentions)*	
CCA action plans are still often missing and/or they are not properly aligned with the biodiversity and climate mitigation strategies, and regulation	Municipal strategy for increasing resilience during floods and droughts—this gives a strong basis for an ambitious stormwater programme, and regulation
*1.2 No municipal-level commitment for requirements and principles (6 mentions)*	
Consistent long-term implementation of stormwater regulations in municipal planning is not possible if the high-level commitment and statements on stormwater management principles and requirements are missing	Approval of a stormwater programme stating stormwater management principles by the city council. This ensures continuity and facilitates communication between different actors
*1.3 No consideration of climate vulnerability in regulation (6 mentions)*	
The regulations for water retention/detention and increase of green spaces are not sufficiently linked to local site characteristics and climate vulnerability analysis, in spite of the potential of the zoning plans to adapt the rules to the zone climate vulnerabilities	Adapt the regulations to the spatialised context and special local characteristics (such as soil type and drainage basin) of the cities in terms of climate vulnerabilities to flood and provision of biodiverse green spaces
*1.4 The lack of binding force of the regulations (4 mentions)*	
Recommendations do not work, requirements are needed	Policies that make the BGF target levels binding
2. Shortcomings of the current stormwater regulations	
*2.1 BGF requirement for different types of land use (3 mentions)*	
In many cities, detached and semidetached houses are exempted from requirements and the BGF requirements are rarely used in industrial or logistics lots	The BGF requirements should be extended especially to industrial and logistics sites, as they are hot spots of stormwater management
*2.2 Unclarity of the requirements in retrofitting sites (2 mentions)*	
Unclarity of the lot area where requirements must be met in retrofitting cases—do they concern the whole lot or just the part that is being developed?	Possibility to combine lots and use BGF targets. This provides flexibility in the planning and allows construction of a stormwater management solution of significant size
*2.3 Unclarity of the (4 mentions)*	
Unclarity of BGF calculation sheets and possibilities to deliberate misinterpretations	Development of a robust calculation basis that does not give place for different interpretations
*2.4 No NbS requirement for water detention (4 mentions)*	
The water detention requirements do not incentivize or impose use of NbS, thus grey solutions are applied	Introduction of regulation that requires that a certain percentage of the required detention space has to be realised using rain gardens
3. Lack of know-how on NbS	
*3.1. Lack of expertise of NbS in building inspection (2 mentions)*	
The building inspection officers lack expertise on NbS and stormwater retention solutions, they are often engineers, but have no expertise on plants and soil, and the ecological aspects of green infrastructure	A building inspector that is specialised in blue–green infrastructure and has expertise for assessing the garden plans and blue–green infrastructure
*3.2. Lack of know-how in planning and construction (6 mentions)*	
Planners and construction companies are lacking know-how for planning and constructing nature-based stormwater solutions. Stormwater planning is usually done by the HPAC engineers, who do not have expertise on NbS, whereas underground stormwater cassettes and large pipes are well known by planners and construction companies	Individual guidance for constructors, property developers and individual house owners. Nation-wide practices in regulation and planning of stormwater systems would allow more cost-efficient development of guidance and training
*3.3. Lack of knowledge of NbS requirements, costs and benefits (5 mentions)*	
There is uncertainty about the actual costs, functionality and maintenance needs of the NbS, and a gap of awareness or calculation of the non-monetary benefits	Systematic collection of examples and experiences and information sharing between cities
4. Organisational and structural challenges	
*4.1. Lack of space in built environment (5 mentions)*	
Lack of space and the prevalence of deck yards are limiting the use of NbS—more information and experiences are needed on NbS for stormwater that can be fitted into densely built environments in Nordic climate	No best practices or recommendations from the interviews or the subsequent workshop
*4.2. No subsequent inspections of installed stormwater system (6 mentions)*	
The building inspection departments lack resources, and on-site inspections are non-existent or done too late—due to the lack of surveillance, the plans are not always realised as stated in the plans	Site visits for planners to forerunner properties with innovative use of GI to increase their motivation, investing on capacity building of building inspection departments
*4.3. Lack of communication (5 mentions)*	
Feedback about the functionality of provisions and regulations from the permitting and realisation phase do not often reach the planners—lack of communication between different departments	Regular communication between the planning department and building inspection department would allow organisational learning and development of the processes from planning to realisation

Interviews noted that assigning stormwater management responsibility to municipalities without clear instructions gives freedom but also causes confusion, leading to inconsistent practices within and between the cities (2.1–2.3). This also hinders the formulation of long-term plans with CCA goals (1.1–1.3). The interviews highlighted that inconsistent interpretations and limitations in building inspection (4.2) are common side effects of unestablished institutional practices in stormwater regulation (1.2, 1.4). Building inspections are typically carried out at the stage where it is no longer possible to fully examine or modify stormwater management solutions (4.2). In worst case, this allows nature-based alternatives to be left out during implementation, despite regulatory requirements during the planning phase. However, neither the interviews nor the building permit analysis clearly indicated whether this occurs in practice. Experts suggested providing training for building inspectors to improve their ability to recognise nature-based structures or their absence through site visits to innovative GI sites.

Interviews also highlighted the lack of communication (4.3) and expertise (3.1–3.3) throughout the planning and implementation processes. Planners often lack knowledge of the actual cost and user experience, despite the availability of such information (3.3). Additionally, the voluntary nature of new practices and the low value of NbS in the regulations reduce the interest and motivation to implement them (1.4). Technical solutions were seen as a familiar and easy solutions with reliability and well-documented implementation and usage. This points to a professional or educational barrier as well as issues in communication, institutional fragmentation and sectoral silos. Combining water management and yard planning is seen difficult because these entities do not communicate during planning. The suggested solutions included regular communication between the planning and inspection departments, systematic collection of case examples, inter-city knowledge sharing, and clearer guidance for inspectors and constructors. These actions would allow organisational learning over the whole process.

Space limitations and the pressure for high-density development of lots are also significant challenges for NbS implementation (4.1). The lack of space and, for example, the prevalence of deck yards limit the use of NbS. In addition, other regulatory requirements, such as recycling facilities, bicycle storage, and rescue ways, compete for limited yard space. Experts were unable to identify potential solutions for the challenges posed by densification. Generally, more information and experience are needed on NbS that can be fitted into densely built environments in the Nordic climate.

### Blue–green factor in promoting nature-based solutions in water management

Grey infrastructure dominates site-level stormwater management. Building permit analysis revealed that the majority of required stormwater management relied on grey solutions, regardless of NbS aims and regulations (Table 4). Of the 39 cases, 56% used large underground detention pipes, 28% used underground storage tanks or cassettes, and 15% used above-surface stone-covered retention structures. Only 13% used NbS, and none relied solely on them, despite the fact that many of the plans mentioned the goal of NbS usage to the largest possible extent. The use of impermeable surfaces varied significantly, with a minimum of 29.4% to a maximum of 97.5% of the area covered with impervious materials (including buildings). The average proportion of green area was 20% of the total area. The BGF alone was used for stormwater regulation in 11 of the 39 cases, whereas in 28 of the cases, the Hule-100 rule was integrated into BGF calculations (Table 4). No significant differences were found in the planning practices between the two approaches.

**Table 4:** Main statistics from BGF calculations of the new construction sites in Turku (*n* = 39). *At some of the sites, Hule-100 and BGF were used together, and at some, only BGF was used

Feature	Total
Mean lot size	5927.9 m^2^
Mean impervious area (% from the total area)	3394 m^2^ (56.5%)
Min impervious area (% from the total area)	506 m^2^ (29.4%)
Max impervious area (% from the total area)	20,219 m^2^ (97.5%)
Mean green area (% from the total area)	1221 m^2^ (20%)
BGF usage/Hule − 100 + BGF*	*n* = 11/*n* = 28
NbS Types of nature-based stormwater detention solutions	Vegetated management structure (not specified), reservoir, raingarden,
Number of sites with nature-based stormwater detention solutions	*n* = 5
Average amount of NbS in the same site	1
Grey stormwater detention solutions	Detention tank/vault, detention pipes, cassette, stone-covered management structure (not specified), excavation
Grey solutions only sites/Grey and NbS sites/NbS sites	*n* = 34/*n* = 5/*n* = 0
Green roof(s)	*n* = 24

**Fig. 5:**
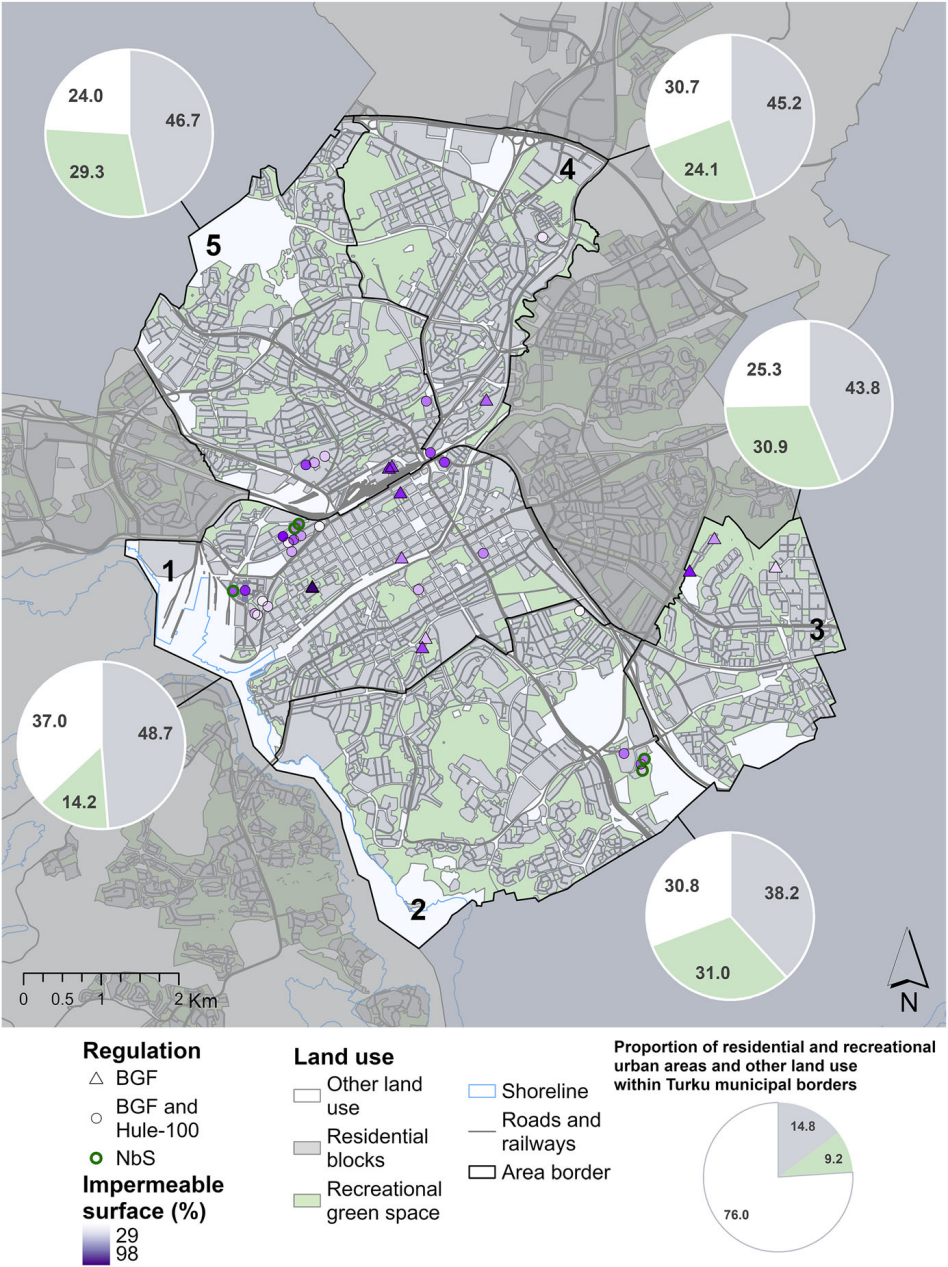
Regulatory methods and the proportion of impermeable surface area from total plot area in analysed building sites. The building permit sites are in five major districts of Turku: 1) City centre, 2) Skanssi, 3) Varissuo, 4) Runosmäki and 5) Länsikeskus districts. The distribution of land use, and particularly the extent of green areas, varies significantly across the districts reflecting the overall difference in the density of urban structure between the areas. Regulatory mechanism does not seem to explain the amount of impermeable surface or NbS use

Building permit sites are in five different districts that vary in land use and population density (Fig. 5). Sites with large percentage of impermeable surfaces seemed to be located mostly in City centre (1) and Länsikeskus (5) areas, but one is also located in the sparser Varissuo (3) area. Simultaneously, sites with more permeable surfaces were evenly distributed across the areas. Interestingly, the sites regulated by BGF only were generally more impervious than those regulated by BGF and Hule-100 together. All NbS were also located at sites using both regulations, suggesting that using current regulations together is the most effective way to promote GI, perviousness, and NbS use in stormwater management.

Building permit documentations from different sites revealed discrepancies and inconsistencies in the BGF calculation sheets and other documents. For example, the areas of different elements differed between documents from the same site. There were also differences between what was considered in which section of the BGF calculations and the elements and solutions mentioned in the planning documents were not aligned with each other. This significantly impacted the performance of the BGF tool and made it more difficult to evaluate regulatory impacts. BGF target value was possible to achieve without incorporating NbS for water management. However, the implemented solutions included unconventional choices, suggesting that these policy instruments have nevertheless diversified the range of solutions used.

## Discussion

Stormwater policy, governance, and regulation in Finland are multilevel and multidisciplinary processes strongly influenced by EU water and environmental policy. However, national stormwater regulations have not been updated to meet the needs of modern climate- and nature-sensitive stormwater management. Consequently, municipalities have created their own regulations and practices. This has led to the fragmentation of regulatory practices, which is a significant barrier to building the capacity of different actors to implement NbS in stormwater management. Effective stormwater management requires well-established policies and governance (Novaes and Marques 2022, 2023). Many other countries have national stormwater policies, regulations, and implementation guidelines, such as Low Impact Development and Sustainable Drainage System policy statements that support natural hydrology and sustainability (Ellis and Lundy 2016; Henstra et al. 2020). Although local mandatory regulations have proven effective in promoting NbS (Mandarano and Meenar 2017; William et al. 2017), previous studies have noted that municipal-level regulations can lead to inconsistencies. Gaps and differences between regulations have caused shortfalls in achieving environmental goals (Jensen et al. 2020). A national consensus is required to establish consistent frameworks, terminology, and goals. This can be achieved through the development of national BMPs. A shared understanding of objectives across municipalities would likely increase the coherence and success of NbS implementations. This study recommends establishing national-level water management goals and national BMPs to address the broader picture of nature-based water management.

Urban areas are particularly challenging due to impervious surfaces, making it essential for actors in these areas to take responsibility for stormwater management (Venuti et al. 2025). This study found that all studied cities have taken steps towards mainstreaming NbS in water management. Most have drafted a stormwater programme to support sustainable stormwater management. These programmes show cities’ ambition and motivation to address common stormwater challenges, such as water quality and quantity issues, and wider environmental issues such as biodiversity loss and climate change. However, this study also demonstrates that political ambition alone is not enough. Local differences in conditions and regulations also shape NbS use, requiring a national and city-level re-evaluation of the goals. Researchers have suggested a flexible and pragmatic approach for stormwater regulators that considers the limitations of individual municipalities (McDonald and Naughton 2019). This could again be achieved through national BMPs or Best Available Techniques supported by CCA strategies that integrate NbS into community-level water management (McDonald and Naughton 2019; Jensen et al. 2020; Corgo et al. 2024). National-level statements and binding NbS and GI agreements would align practices more closely with policy goals by clarifying municipal regulations.

Municipal-level regulations have proven effective in encouraging investments in NbS (Mandarano and Meenar 2017) and in addressing the challenges posed by private land ownership (Johns 2019; Venuti et al. 2025). However, several well-known barriers were identified in this study as still existing bottlenecks in the regulatory frameworks. Lack of enforcement and inconsistent practices hinder NbS implementation. Regulations were applied with different spatial extensions, restrictions, and enforceability. Both existing regulations, BGF and Hule-100, have benefits and drawbacks. The common opinion was that both regulations can guide stormwater management to sustainable direction. Th BGF promotes green elements, and the latest improvements to Hule-100 have included nature-based water management elements. However, the BGF’s inflexibility, lack of NbS, and/or dysfunctional valuation of NbS limit its impact. For example, while the BGF can include a binding target value, it can be met without implementing NbS. The guidance and categories also remain unclear and underdeveloped; for instance, a single BGF element can fulfil the criteria for multiple categories. Flexibility and adaptability can be strengths. However, exemptions and self-selection of elements weaken enforcement and can allow NbSs to be bypassed entirely. Further research is needed to collect best practices and understand how differences in stormwater regulation between cities influence the use of NbS in water management. This study indicates that BGF and Hule-100 are the most effective when combined, highlighting the need for integrated planning regulations that embrace the multifunctionality of green infrastructure.

The BGF has previously been seen as a promoting factor for NbS (Venuti et al. 2025). In this study, BGF was valued for its transparency and ability to promote nature-based planning, but in the practical review, its value for increasing NbS implementations was not clear. Despite the use of BGF and goals for prioritising NbS, these solutions appeared in only a few new construction sites and only covered a fraction of the required detention volumes. Overall, the sites were densely built included many impermeable surfaces. However, discrepancies in the planning documents and BGF calculation created uncertainty in the analysis. Such errors and the use of grey solutions despite regulations promoting urban greening may indicate that the regulations are unclear or unsuitable. This can stem from the fact that BGF was not originally designed to regulate stormwater management, even though it is increasingly used for this purpose. BGF sites may feature more GI, but detention often relies on grey infrastructure. To profoundly understand the reasons for the sparse use of NbS, observed barriers and bottlenecks can be revisited. Factors indicating a lack of knowledge, such as a small selection of NbS and a large selection of technical solutions, may have a greater influence than the regulation method itself. Further investigation is needed to fully understand the reasons for the current planning decisions.

An increase in knowledge and cooperation have emerged as central goals in stormwater programmes. However, the interviews revealed major bottlenecks, such as lack of knowledge and know-how, together with institutional bottlenecks, such as unambiguous practices and lack of communication. These findings are consistent with the barriers identified in prior research (Johns 2019; Sarabi et al. 2019). Supervision and communication are needed to ensure efficient local and regional stormwater management (McDonald and Naughton 2019). Although some barriers were already recognised in the city stormwater plans, this study reveals their ongoing existence as bottlenecks in all levels of stormwater planning. This may be due to path dependency, sectoral silos, hesitance, and lack of institutional commitment, making the change slow and difficult (Henstra et al. 2020). Integrated and cooperative planning has also been considered as time-consuming and difficult in previous research (Santos et al. 2019). Achieving a common understanding and cooperative planning requires abandoning sectoral thinking, defining responsibilities, and systematically sharing information. Municipal-level and cross-municipal pilot projects to share knowledge and positive experiences are recommended, as this has been seen as a promoting factor for NbS.

City development trends are also a challenge for NbS. Our building permit analysis suggests that strategic development goals may influence the use of GI and NbS more than current regulations. Previous research has noted that regional history shapes current development paths, while internal dynamics and economic forces ultimately determine the trajectory of urban development (Mandarano and Meenar 2017). This study supports these findings: regulations alone cannot steer the development of a city or alter the inherent characteristics of an area. Strategies and agreements, such as the land use, housing, and transport (MAL) agreements promoting urban densification, may overrule local regulatory aims.

Although municipal stormwater financing was not a central theme in this study, resource availability has clearly impacted the quality and results of stormwater planning and strategic work. The results of this study indicate that city size affects the ambition of future goals and implementation, possibly resulting in smaller cities falling behind in NbS implementations. Resources have been noted to have a significant impact on the amount and quality of stormwater instructions and communication (McDonald and Naughton 2019). Further studies on what motivates private developers to implement NbS could improve regulatory support mechanisms and the allocation of limited resources. Stormwater fees can motivate private landowners to implement NbS if fees are linked to the site's capacity to retain water on-site, thus providing a clear financial incentive for property owners. The potential of this approach was modelled by William et al. (2017), who found it to be a promising strategy. The findings of our study further support the use of incentive mechanisms to promote NbS at the property level. Currently, NbS implementations on private properties are the landowners’ responsibility, yet these efforts receive no compensation or incentives, despite their contribution to reducing pressure from municipal stormwater systems.

This study identified still existing institutional and organisational bottlenecks, engagement and uncertainty bottlenecks, educational, training, and knowledge bottlenecks, city structure bottlenecks, and insufficient regulation. There is also a clear gap between regulations and practice. These findings align with the known barriers in the literature. Stormwater management, NbS, and GI experience these barriers in policy, decision-making, and practical instances (Cohen-Shacham et al. 2019; Johns 2019; Santos et al. 2019; Sarabi et al. 2019). Importantly, many possible solutions were identified at the grassroots level by the practitioners. Experts suggested stronger municipal regulation, communication, engagement, education, and knowledge, stating the obviousness and visibility of the issues for the stakeholders themselves. Knowledge needs to be increased top-down and bottom-up: planners must improve tools, while constructors need training in NbS and in regulatory frameworks. These transboundary actions have been identified as crucial for the successful implementation and uptake of NbS (Sarabi et al. 2019).

Regulatory tools can bridge the gap between policy and practice by contextualising environmental goals, but their design and implementation must consider multiple factors. This study found that current regulations are not highly effective in promoting NbS. However, based on the document analysis, interviews, and building permit analysis, it is possible to successfully implement NbS requirements for stormwater regulation. Currently, the vagueness of the NbS definition in regulations makes it harder for grassroots-level stakeholders, such as building companies, to recognise NbS as a viable option. The selection of NbS needs to be re-evaluated, and NbS must be emphasised in the regulations. NbS should be based on clear and widely accepted key principles, and the existing concepts need to clarify their added value compared to alternatives (Cohen-Shacham et al. 2019). One of the recognised NbS strengths is the possibility of implementing NbS and grey solutions together (Cohen-Shacham et al. 2019). Full retention through NbS can be difficult in dense urban settings. Flexible, clearly defined, and enforceable regulations are required. Municipal-level stormwater regulations can be effective (Mandarano and Meenar 2017; William et al. 2017), but as emphasised, regulation alone cannot solve all bottlenecks.

## Conclusions

This study investigated municipal-level stormwater policy, regulation, and implementation to understand the current state of stormwater planning and the basis for implementing NbS. While previous studies have identified challenges in decision-making, it remains poorly understood whether and how regulations increase NbS adoption on privately owned land.

The findings of this study suggest that municipal stormwater programmes often set ambitious goals for NbS implementation. However, the absence of a national-level consensus and differing local conditions have resulted in inconsistent regulations and unestablished practices, which hinder implementation of NbS on private land. The main bottlenecks are institutional weaknesses, knowledge and communication gaps, and uncertainty, affecting all stages of planning and implementation. Discrepancies in planning regulations and a limited understanding of NbS functionality and cost further reduce the motivation for their application.

Ensuring NbS implementation in stormwater management requires strong motivation and ambition. A national stormwater policy that integrates NbS as a central approach to address both quantity and quality challenges, alongside nation-wide BMPs and standardised municipal regulations, would form an ideal foundation. Enhancing implementation on private land will require planning regulations that mandate NbS elements, recognise their benefits, and enable flexible integration with grey infrastructure. Beyond regulation, supportive measures are critical, including targeted education and training, demonstrative pilot projects, monitoring and inspections, and financial incentives, such as runoff-based stormwater fees. Such supportive mechanisms can build awareness, practical capacity, and tangible motivation for implementing NbS.

## Supplementary Information

Below is the link to the electronic supplementary material.

Supplementary file1 (PDF 111 KB)
