# Supplementary material for: Policy and regulatory measures supporting the implementation of nature-based solutions in urban stormwater management of private properties: Insights from Finland
Source: Ambio. 2025 Sep 27;55(4):903–20. doi: 10.1007/s13280-025-02253-2 (PMC12960866; doi:10.1007/s13280-025-02253-2)
Supplement: Supplementary file 1 — Supplementary file1 (PDF 111 KB) [file 13280_2025_2253_MOESM1_ESM.pdf]

Ambio

Supplementary Information
This supplementary information has not been peer reviewed.

Title: **Policy and Regulatory Measures Supporting the Implementation of Nature-Based Solutions in Urban Stormwater Management of Private Properties: Insights from Finland**

Table S1. The names and sources of the strategies and stormwater programmes analysed in this research.

	Document name	***Provider***	***Available at***
Stormwater programmes	Helsingin kaupungin hulevesiohjelma	City of Helsinki	https://www.hel.fi/static/liitteet/kaupunkiymparisto/julkaisut/julkaisut/julkaisu-03-18.pdf&ved=2ahUKEwiI0oiP7bWIAxWIHBAIHfC4BtIQFnoECAgQAQ&usg=AOvVaw3mmsEgdIH4xKyqxv4CXNQw
	Hulevesiohjelma	City of Lahti	https://www.lahti.fi/asuminen-ja-ymparisto/ymparistonsuojelu-ja-valvonta/ympariston-tila/hulevedet-ja-niiden-hallinta/
	Tampereen kaupungin hulevesiohjelma ja valuma-alueselvitys 2023-2030	City of Tampere	https://www.tampere.fi/asuminen-ja-rakentaminen/rakenna-ja-korjaa/hulevedet#paragraph-104038
	Turun kaupungin hulevesiohjelma 2016-	City of Turku	https://www.turku.fi/asuminen-ja-ymparisto/ymparisto/vesiensuojelu/hulevedet
City strategies	Kaupunkistrategia Turku 2030-luvulla	City of Turku	https://www.turku.fi/kaupunkistrategia-turku-2030
	Tehty Kestäväksi, Lahti 2030, Lahden kaupungin strategia 2030	City of Lahti	https://www.lahti.fi/kaupunki-ja-paatoksenteko/kaupungin-strategia/
	Kasvun paikka – Helsingin kaupunkistrategia 2021–2025	City of Helsinki	https://www.hel.fi/fi/paatoksenteko-ja-hallinto/strategia-ja-talous/strategia
	Tampereen strategia 2030 - Tekemisen kaupunki	City of Tampere	https://www.tampere.fi/tampereen-strategia
	Yhdessä paras Kaarina, Kaarina-strategia 2023–2030	City of Kaarina	https://kaarina.fi/fi/strategia#4b7e68d5

Table S2. Interview questions of the key informant interviews. The questions varied slightly between the expert groups.

Group	***Questions***
City administrators	• Do you think the blue-green factor and the related calculation tool were a working regulatory tool? What has been functional and good about the tool? What has not worked and what are the challenges involved?<br>• Do you think the Hule-100 were a working regulatory tool? What is workable and good? What has not worked and what are the challenges involved?<br>• Does the use of the blue-green factor cause additional costs or savings in the construction of the property? What are these costs made up of? And same question about the use of hule-100?<br>• Do you think the blue-green factor or hule-100 regulation should be developed? If so, how?<br>• What do you think, why there are less nature-based stormwater solutions implemented compared to technical solutions?<br>• Do you know any other regulatory measures related to the qualitative or quantitative management of stormwater?
Building inspectors	• Do you think the blue-green factor and the related calculation tool were a working regulatory tool? What has been functional and good about the tool? What has not worked and what are the challenges involved?<br>• Do you think the Hule-100 were a working regulatory tool? What is workable and good? What has not worked and what are the challenges involved?<br>• Does the use of the blue-green factor cause additional cost or savings to the city? What are these costs made up of? And same question about hule-100?<br>• Have there been any complaints about the blue-green factor or the hule-100 regulatory? What have these complaints been about and from whom have they come?<br>• Do you think the blue-green factor or hule-100 regulation should be developed? If so, how?<br>• Is the implementation of regulatory instruments supervised? If so, how? At what point in the process?<br>• What do you think, why there are less nature-based stormwater solutions implemented compared to technical solutions?
Zoning planners/Land use planners	• Do you think the blue-green factor and the related calculation tool were a working regulatory tool? What has been functional and good about the tool? What has not worked and what are the challenges involved?<br>• Do you think the Hule-100 were a working regulatory tool? What is workable and good? What has not worked and what are the challenges involved?<br>• How has the blue-green factor been developed during its use? How about the hule-100 regulatory?<br>• Should those be further developed? If so, how?<br>• Do you use any other regulatory measures related to the qualitative or quantitative management of stormwater?<br>• Does the use of the regulatory cause additional cost or savings to the city? What are these costs made up for?
